# Establishment and validation of a guinea pig model for human congenital toxoplasmosis

**DOI:** 10.1186/s13071-021-04890-4

**Published:** 2021-08-06

**Authors:** Thomas Grochow, Britta Beck, Zaida Rentería-Solís, Gereon Schares, Pavlo Maksimov, Christina Strube, Johannes Seeger, Lisa Raqué, Reiner Ulrich, Arwid Daugschies, Simone A. Fietz

**Affiliations:** 1 Institute of Veterinary Anatomy, Histology and Embryology, Faculty of Veterinary Medicine, University of Leipzig, Leipzig, Germany; 2 Institute of Parasitology, Faculty of Veterinary Medicine, University of Leipzig, Leipzig, Germany; 3 National Reference Laboratory for Toxoplasmosis, Institute of Epidemiology, Friedrich-Loeffler-Institut, Federal Research Institute for Animal Health, Greifswald-Insel Riems, Germany; 4 Institute for Parasitology, Centre for Infection Medicine, University of Veterinary Medicine Hannover, Hannover, Germany; 5 Veterinary Practice Raqué, Leipzig, Germany; 6 Institute of Veterinary Pathology, Faculty of Veterinary Medicine, University of Leipzig, Leipzig, Germany

**Keywords:** Congenital toxoplasmosis, *Toxoplasma gondii*, Guinea pig, Animal model, Oocysts infection, Stage of gestation, Infection dose, Predilection site, Brain lesion

## Abstract

**Background:**

*Toxoplasma gondii* is an obligate intracellular parasite with a worldwide distribution. Congenital infection in humans and animals may lead to severe symptoms in the offspring, especially in the brain. A suitable animal model for human congenital toxoplasmosis is currently lacking. The aim of this study is to establish and validate the guinea pig as a model for human congenital toxoplasmosis by investigating the impact of the *T. gondii* infection dose, the duration of infection and the gestational stage at infection on the seroconversion, survival rate of dams, fate of the offspring, *T. gondii* DNA loads in various offspring tissues and organs and the integrity of the offspring brain.

**Methods:**

Pregnant guinea pigs were infected with three different doses (10, 100, 500 oocysts) of *T. gondii* strain ME49 at three different time points during gestation (15, 30, 48 days post-conception). Serum of dams was tested for the presence of *T. gondii* antibodies using immunoblotting. *T. gondii* DNA levels in the dam and offspring were determined by qPCR. Offspring brains were examined histologically.

**Results:**

We found the survival rate of dams and fate of the offspring to be highly dependent on the *T. gondii* infection dose with an inoculation of 500 oocysts ending lethally for all respective offspring. Moreover, both parameters differ depending on the gestational stage at infection with infection in the first and third trimester of gestation resulting in a high offspring mortality rate. The duration of infection was found to substantially impact the seroconversion rate of dams with the probability of seroconversion exceeding 50% after day 20 post-infection. Furthermore, the infection duration of dams influenced the *T. gondii* DNA loads in the offspring and the integrity of offspring brain. Highest DNA levels were found in the offspring brain of dams infected for  ≥ 34 days.

**Conclusion:**

This study contributes to establishing the guinea pig as a suitable model for human congenital toxoplasmosis and thus lays the foundation for using the guinea pig as a suitable animal model to study scientific questions of high topicality and clinical significance, which address the pathogenesis, diagnosis, therapy and prognosis of congenital toxoplasmosis.

**Graphical abstract:**

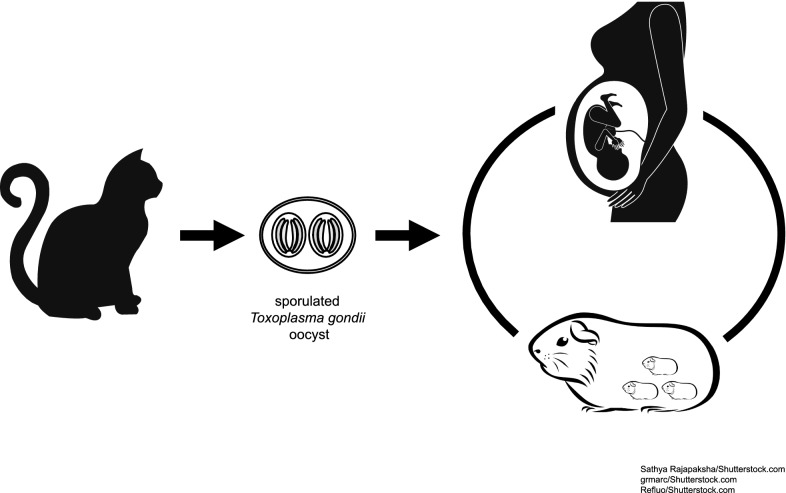

**Supplementary Information:**

The online version contains supplementary material available at 10.1186/s13071-021-04890-4.

## Background

Toxoplasmosis is a zoonosis caused by *Toxoplasma gondii*, an obligate intracellular parasite with a worldwide distribution [1–3]. The global seroprevalence for *T. gondii* in adults is approximately 33% [4, 5]. The global incidence of *T. gondii* infection during pregnancy is 1.5% for women of childbearing age [5]. Congenital *T. gondii* infections are of clinical and economic importance in pets and livestock, especially in small ruminants [6–8]. The definitive hosts of *T. gondii* are cats and other Felidae; all warm-blooded animals are intermediate hosts. *T. gondii* passes through several developmental stages in its life cycle [9, 10]. Sexual reproduction of the parasite occurs in the intestinal epithelium of the definitive host. The resulting oocysts are shed in the feces [11]. Oral infection of an intermediate host results in rapid asexual multiplication of the acute (tachyzoite) stage [10, 12]. Tachyzoites can reach and infect all tissues of the host via the bloodstream, lymphatic system and peritoneal fluid. Due to the reaction of the immune system, the parasite is pushed back into the chronic stage, and intracellular tissue cysts containing bradyzoites are formed about 10–14 days after infection. Bradyzoites have a reduced metabolism, and cysts may persist for life in the organism, especially in the brain, retina, and skeletal and cardiac muscles [10, 13–16]. Main routes of infection of the intermediate host are ingestion of food or water contaminated with sporulated oocysts and raw or insufficiently treated meat containing cysts. Given that the parasite crosses the placental barrier, *T. gondii* may be transmitted to the embryo/fetus during maternal parasitemia after a primary infection of the mother [1, 3, 17]. In adult healthy and immunocompetent humans, initial infection is usually asymptomatic. However, congenital infection may lead to severe symptoms in the offspring. The pathological changes occur particularly in the developing central nervous system [9, 18]. In this regard, the clinical picture in humans depends on the time of infection during pregnancy, i.e. the earlier the time of infection, the more severe the symptoms caused by *T. gondii*. Infection in the first trimester may cause abortion. Microcephaly, retinochorioiditis and mental retardation are classic signs of a relatively severe course and may indicate infection in the second trimester. When infection occurs in the third trimester of pregnancy, cerebral calcifications and hepatosplenomegaly predominate [19, 20].

A suitable animal model for human congenital toxoplasmosis is currently not available. Mouse and rat appear to be less suitable because of their relatively short gestation period of approximately 20–22 days [21–23]. Moreover, both species are—in contrast to humans—primary altricial mammals and are therefore characterized by a low degree of maturation at the time of birth and the development of a hemotrichorial placenta, in which three layers of chorionic epithelium separate the maternal blood from the fetal capillaries [24]. Large domestic mammals such as ruminants and pigs are precocial mammals. However, as ruminants and pigs develop an epitheliochorial placenta, in which a single-layer chorionic epithelium borders the intact wall of the endometrium and underlying maternal capillaries, they appear to be less suitable as animal models for studying congenital infections [24]. The guinea pig is a precocial mammal, whose brain, similar to that of humans, is characterized by a relatively high degree of maturity at birth [25]. In analogy to the human cortex, neurogenesis in the guinea pig neocortex mainly occurs in the second trimester of gestation [26]. Moreover, it shows great similarities with humans regarding the placentation. Similar to humans, the guinea pig exhibits a haemomonochorial placenta, in which a single-layer, syncytial chorionic epithelium is in direct contact with the maternal blood. Furthermore, in both species, progesterone is produced by the placenta during pregnancy and the sexual cycle is characterized by a cyclically recurring estrus [27–29]. In addition, the guinea pig has a relatively long gestation period of approximately 66 days. Due to these characteristics, the guinea pig has been considered a suitable model animal for the study of human congenital toxoplasmosis [21, 22, 30]. Depending on the *Toxoplasma* strain, dose and mode of infection, toxoplasmosis in adult guinea pigs can be asymptomatic, but may also lead to myocarditis, myositis, encephalitis, pneumonia and hepatitis [21, 31, 32].

The aim of this study is to establish and validate the guinea pig as a model animal for human congenital toxoplasmosis by investigating the impact of the *T. gondii* infection dose, the duration of infection and the gestational stage at infection on the seroconversion rate, the survival rate of the dams, the fate of the offspring, the *T. gondii* DNA loads in various offspring tissues and organs and the integrity of the offspring brain. This study provides the basis for the proposed use of the guinea pig as an animal model for the investigation of scientific aspects of high medical importance, particularly the pathogenesis of congenital toxoplasmosis in humans.

## Methods

### Animals

Female Dunkin Hartley guinea pigs (*n* = 30) were obtained from Charles River Laboratories (Ecully, France) and housed in the animal-care facility of the Institute of Parasitology, Faculty of Veterinary Medicine, University of Leipzig, Leipzig, Germany. Animals were kept in groups of two in 50 × 70 × 50 cm wire mesh cages. Water supplemented with 400 mg/l ascorbic acid, standard diet pellets (Altromin Spezialfutter, Lage, Germany) and fresh vegetables were provided ad libitum. Room temperature was kept at 19–25 °C, and relative humidity averaged 55% (± 10%). Light/dark cycle was set to 12:12.

### Mating and pregnancy examination

Females (*n* = 30) were mated at the Institute of Parasitology, Faculty of Veterinary Medicine, University of Leipzig. Prior to breeding, their sexual cycle was synchronized as described previously [33]. Briefly, females were orally administered Altrenogest (Regumate® Equine 2.2 mg/ml, MSD Tiergesundheit, Unterschleißheim, Germany) at 0.22 mg/kg body weight once a day for 15 days. Two days after the end of treatment, four females were housed together with one breeding ram for a total of 4 days to initiate natural mating. Successful pregnancy was determined by ultrasound using GE Logiq 400 CL (pet mode, 7–10 MHz sample, GE Healthcare, Solingen, Germany). As a result, the day of successful mating is considered as day 0 ± 2 of pregnancy. During ultrasonographic examination, the reproductive system including ovary and uterus wall of all dams was examined. In addition, the vagina of all dams was examined by inspection.

### Serological investigation

Blood samples of dams were collected from lateral saphenous vein immediately before infection and by intracardiac puncture at the respective end of trial. Samples were centrifuged (2500×*g*, 10 min, 4 °C); serum was collected and analyzed using immunoblotting against *T. gondii* surface antigen p30 (SAG1) as described previously [34] with the modification that, instead of the peroxidase conjugated anti-mouse IgG, a peroxidase conjugated anti-guinea pig IgG (H + L) (Jackson Immunoresearch Laboratories, West Grove, USA) was used.

### Infection

*Toxoplasma gondii* oocysts (Strain ME49) were obtained from the Institute of Parasitology, University of Veterinary Medicine Hannover, Germany. Seronegative cats were fed with meat supplemented with brain, muscle, liver and spleen of guinea pigs chronically infected with *T. gondii*. The presence of the parasite in the brain was confirmed by squash histology. Cat feces were purified as described previously [35], and isolated oocysts were stored in 2% sulfuric acid at 4 °C for 1.5–4 months. Immediately before infection, the oocyst suspension was neutralized with 1 M sodium hydroxide solution, and sporulated oocysts were quantified using a Neubauer-Improved haemocytometer (Paul Marienfeld, Lauda-Königshofen, Germany). Oocysts were suspended in 500 µl phosphate-buffered saline solution, and the suspension was applied orally using a 16 G buttoned cannula (Henry Schein Dental, Langen, Germany).

Guinea pigs were randomly divided into a control group (*n* = 3) and three different infection groups: low- (*n* = 9), medium- (*n* = 9) and high-dose group (*n* = 9). Dams of the low-dose group were administered 10 oocysts, dams of the medium-dose group were given 100 oocysts, and dams of the high-dose group were administered 500 oocysts on gestation day 15 (*n* = 3), 30 (*n* = 3) or 48 (*n* = 3), respectively. Animals of the control group were given 500 µl phosphate-buffered saline solution only. All *T. gondii* infections were carried out in parallel.

### Euthanasia and dissection

Dams and their corresponding litter were killed by intraperitoneal injection of 500 mg/kg pentobarbital sodium immediately after birth, abortion, resorption or upon the appearance of severe neurological or other clinical symptoms, i.e. ataxia, nystagmus, stupor, somnolence or anorexia, ascites, weight loss of 15% in 2 days, severe pain sensations including salivation, teeth grinding, whimpering and lack of desire to move (Additional file 1: Table S1). Maternal tissue samples were taken from liver, spleen and heart. Offspring tissue samples were taken from liver, spleen, brain, heart and quadriceps femoris muscle and frozen at − 80 °C until further use. Up to 600 mg of tissue was sampled per location. If the entire tissue or organ weighed more, samples were taken randomly from different regions. The brain was removed, and the hemispheres were separated. One hemisphere was used for qPCR; the other one was processed for histological examination.

### Histopathological examination

Brains were fixed in 4% paraformaldehyde for 3 days and cut coronally into five divisions of similar thickness with a scalpel. Each brain division was embedded in paraplast and cut coronally to sections of 1 µm thickness using a microtome (HM 400, Microm, Berlin, Germany). First, one section per division was stained with hematoxylin-eosin, and the number of lesions was counted in the entire section. If a lesion was found, the consecutive section was examined for the presence of microglia by immunohistochemistry using an antibody for Iba1 (2-MI004-10, Quartett, Berlin, Germany). Hematoxylin-eosin staining and immunohistochemistry were performed as described previously in [36–38]. An Olympus BX46 light microscope (Olympus, Shinjuku, Japan) equipped with an Axiocam 208 color digital camera (Zeiss, Oberkochen, Germany) and ZEN 2.6 (Zeiss, Oberkochen, Germany) imaging software was used to examine histological sections.

### Tissue homogenization and DNA extraction

Tissue samples of dams and corresponding offspring were processed using NucleoMag-Tissue kit (Macherey-Nagel, Düren, Germany). The manufacturer's instructions were adjusted as follows: Tissue samples up to a maximum of 600 mg were incubated with lysis buffer (T1) supplied with the NucleoMag-Tissue kit (Macherey*–*Nagel, Düren, Germany) at 1:2 ratio. Two stainless steel balls (diameter 6 mm, TIS Wälzkörpertechnologie, Gauting, Germany) were added, and the sample was homogenized using a TissueLyser II (Qiagen, Hilden, Germany) at 3000 Hz for 60 s. In a next step, Proteinase K solution provided by the NucleoMag-Tissue kit was added to the tissue buffer mixture at 1:20 ratio and the suspension incubated overnight at 56 °C. All further extraction steps were carried out in a King Fisher Flex (Thermo Scientific, Waltham, MA, USA) according to the NucleoMag-Tissue kit manufacturer's instructions.

### Quantitative PCR

The 529-bp repeat element, characterized by high specificity ad sensitivity for detection of *T. gondii*, was used as a target in a probe-based qPCR [39, 40]. For dams and corresponding offspring, quantitative PCR was carried out as follows: primers and probes (MWG-Biotech, Ebersberg, Germany) were applied as described previously [41] according the assay protocol "Toxo529REP PCR" (Table 1). An internal control according to [42] was used for inhibition detection as described previously [43] (Table 1). If a negative result was found to be due to PCR inhibition, the respective data were excluded from the data analysis. PCR reactions were carried out in a CFX96 cycler (Biorad Laboratories, Kabelsketal, Germany) using PerfeCTa MultiPlex qPCR ToughMix, (VWR International, Dresden, Germany) and 10 µl of purified DNA. PCR conditions were as follows: 2 min at 50 °C followed by 10 min at 95 °C (initial denaturation). This was followed by 55 cycles, each consisting of 15 s at 95 °C (denaturation) and 1 min at 60 °C (hybridization and elongation). All qPCR results were evaluated using the CFX manager software version 1.6 (Biorad Laboratories, Kabelsketal, Germany). PCR results with a quantification cycle (Cq) ≥ 40 were considered as negative and stated as Cq = 40.

**Table 1:** Primers, probes and their final concentrations in *Toxoplasma gondii* real-time PCR assay

Assay	Names of primers and probes	Sequences of primers and probes 5′–3′	Probe labelling	Final concentration	References
Toxo529REP PCR	TalF	TGG TTG GGA AGC GAC GAG AG		800 nM	[41]
	TalR	CAT CAC CAC GAG GAA AGC GTC		800 nM	[41]
	TalP1_FAM	TGT CGT GCC AGC TGC ATT A	5′-FAM, 3′-BHQ1	200 nM	[41]
Internal control PCR, IC2 PCR	EGFP1-F	GAC CAC TAC CAG CAG AAC AC		500 nM	[42]
	EGFP2-R	GAA CTC CAG CAG GAC CAT G		500 nM	[42]
	EGFP1-Hex	AGC ACC CAG TCC GCC CTG AGC A	5′-HEX, 3′-BHQ1	160 nM	[42]

### Statistical analysis

Statistical analysis was performed using Graphpad Prism 9.0.0 (GraphPad Software Inc., San Diego, CA, USA) software. Normal distribution of all parameters was tested by Anderson-Darling test and D'Agostino and Pearson test. Since none of the data obtained by qPCR analysis and histopathological examination were normally distributed, Mann-Whitney test (for comparison of two groups) and Kruskal-Wallis test (for comparison of more than two groups) were applied for group comparison followed by Dunn's *post hoc* test. For regression analysis, data were analyzed by the method of least squares. For Kaplan-Meyer survival curves, Gehan-Breslow-Wilcoxon test was used to determine statistically significant differences between the different survival curves. *P* values < 0.05 were considered statistically significant. Significance levels were split further according to **P < 0.01, ***P < 0.001, ****P < 0.0001.

## Results

### Fertility and pregnancy examination

A total of 83% (25/30) of dams became pregnant upon first mating. During the ultrasonographic and gynecological examination, no abnormalities were found in either the pregnant or non-pregnant animals. All remaining dams became pregnant after the second synchronization of the sexual cycle. The interval between the first mating and the start of the second cycle synchronization was at least two cycles (32 days).

### Impact of the infection dose and infection duration on the seroconversion

**Fig. 1:**
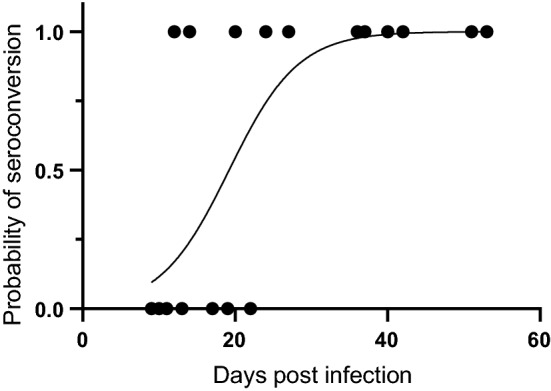
Impact of the infection duration on the seroconversion. Probability of seroconversion of a dam in relation to the duration of infection. Duration of infection was defined by the time between the day of infection and the day of end of observation (day of death, euthanasia, delivery or abortion). Logistic regression analysis showed a significant correlation between both parameters analyzed (*P* < 0.001, R² = 0.57).

All blood samples of the control and infection groups collected prior to infection and all blood samples of the control group collected at the end of the trial were seronegative using immunoblotting against a *T. gondii* p30 surface antigen (Table 2). Of the 27 dams of the infection groups, 17 (65%) blood samples obtained at the respective end of the trial were seropositive. In infected but seronegative dams, the mean duration of infection, i.e. the time between inoculation and the respective end of the trial, was markedly shorter, i.e. 14 days, compared to that of seropositive dams, i.e. 37 days (Table 2). Moreover, logistic regression revealed a significant (Additional file 2: Table S2) relationship between the probability of seroconversion and the duration of infection (Fig. 1). During the first 10 days of infection, the probability of seroconversion is < 25%, rising up to 50% on day 20 of infection and reaching 90% on day 29 of infection (Fig. 1). In nine (of 10) dams, in which no seroconversion was observed, *T. gondii* was directly detected in at least one maternal organ using qPCR (Table 2). As *T. gondii* in the remaining seronegative dam was not detected by qPCR in any of the maternal and offspring organs analyzed (Table 2, no. 1), the infection of this dam was considered unsuccessful, and the dam and its offspring were excluded from the further analysis of this study.

**Table 2:** Overview of the individual litters of guinea pig dams inoculated with *Toxoplasma gondii* oocysts or serving as controls. Each line represents a single litter

Animal number	Inoculation dose^a^	Day of inoculation^b^	Duration of trial^c^	Litter size^d^	Course of pregnancy	Serology mother^e^	qPCR mother^f^	Type of offspring^g^	Offspring included^h^	qPCR of the descendants^i^
1	10	15	51	3	Birth 3 asymptomatic	−	−	N	0	− |−| −
2	10	15	27	(5)	Deceased, autolysis of fetuses	+	+	F	0	N/A
3	10	15	51	0	Resorption	+	+	N/A	N/A	N/A
4	10	30	22	1	Abortion	−	+	F	1	+
5	10	30	42	3	Birth 1 asymptomatic, 2 dead	+	+	N	1	+| +|+
6	10	30	42	4	Birth 3 asymptomatic, 1 dead	+	+	N	1	+| +| +|+
7	10	48	24	2	Birth 0 asymptomatic, 2 dead	+	+	N	1	+|+
8	10	48	19	4	Birth 0 asymptomatic, 4 dead	−	+	N	1	+| +| +|+
9	10	48	17	4	Birth 0 asymptomatic, 4 dead	−	+	N	1	+| +| +|+
10	100	15	51	0	Resorption	+	+	N/A	N/A	N/A
11	100	15	51	0	Resorption	+	+	N/A	N/A	N/A
12	100	15	51	0	Resorption	+	+	N/A	N/A	N/A
13	100	30	36	4	Birth 4 asymptomatic, 0 dead	+	+	N	1	+| +| +|+
14	100	30	36	2	Birth 0 asymptomatic, 2 dead	+	+	N	1	+|+
15	100	30	40	3	Birth 3 asymptomatic, 0 dead	+	+	N	1	+| +|+
16	100	48	11	2	Abortion	−	+	F	1	+|+
17	100	48	10	3	Abortion	−	+	F	1	+| +|+
18	100	48	20	4	Birth 4 living and malformed, 0 dead	+	+	N	1	+| +| +|+
19	500	15	51	0	Resorption	+	+	N/A	N/A	N/A
20	500	15	14	0	Deceased, resorption	+	+	N/A	N/A	N/A
21	500	15	51	(1)	Birth 0 asymptomatic, 1 dead	+	+	N	0	N/A
22	500	30	37	(1)	Abortion, autolysis of fetus	+	+	F	0	N/A
23	500	30	17	0	Euthanasia due to 20% weight loss, fetal absorption	−	+	N/A	N/A	N/A
24	500	30	13	2	Euthanasia due to CNS-symptoms	−	+	F	1	+| −^j^
25	500	48	9	3	Deceased	−	+	F	0	− |−| −^k^
26	500	48	9	4	Abortion	−	+	F	1	+| +| +|+
27	500	48	12	1	Abortion	+	+	F	1	+
28	0	0	66	1	Birth 1 asymptomatic, 0 dead	−	−	N	1	−
29	0	0	66	3	Birth 3 asymptomatic, 0 dead	−	−	N	1	−
30	0	0	66	4	Birth 4 asymptomatic, 0 dead	−	−	N	1	−

^a^Number of sporulated oocysts, ^b^assumed day of pregnancy, ^c^duration from inoculation to birth, abortion or euthanasia. ^d^In the bracketed litters, all fetuses were highly autolytic and were therefore excluded from further analyses. ^e^“ + ” represents a positive result, “-” a negative result, ^f^“ + ” represents a positive result, “-” a negative result, ^g^“N”: neonatal, “F”: fetal, ^h^“1”: included, “0”: not included, ^i^“ + ” represents a positive result, “-” a negative result for each individual offspring. ^j^Only the positive embryo was included in further analyses. ^k^The liver of the mother showed a positive result. These three fetuses were not included in the further examinations

When analyzing the effect of the dose of infection on the probability of seroconversion, no significant relationship was observed using logistic regression (data not shown; Additional file 2: Table S2).

### Impact of the gestational stage at infection and the infection dose on the survival rate of the dams

**Fig. 2:**
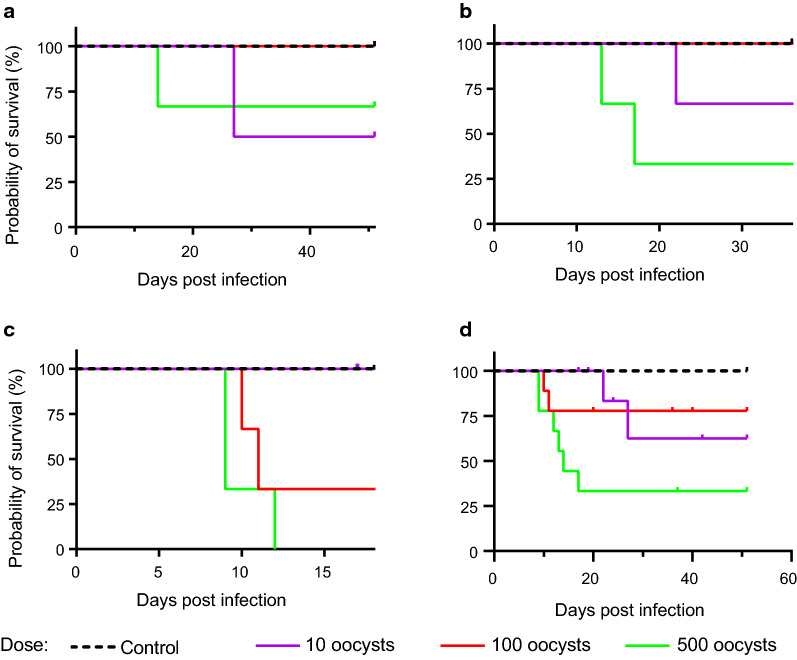
Impact of the infection dose on the dam survival rate. Kaplan-Meier survival rate of dams of the control and different infection groups. Animals were infected with 10 (purple solid line), 100 (red solid line) and 500 (green solid line) sporulated *T. gondii* oocysts on gestation day 15 (**a**), 30 (**b**) and 48 (**c**). Animals of the control group (black dashed line) were administered PBS. For each dam, the day of death, euthanasia, delivery or abortion was recorded. Merged data of (**a–c**) are shown in (**d**). Statistically significant differences between groups were as follows: (**c**) *P* < 0.05, comparing control and 500; (**d**) *P* < 0.05, comparing 10 and 500

**Fig. 3:**
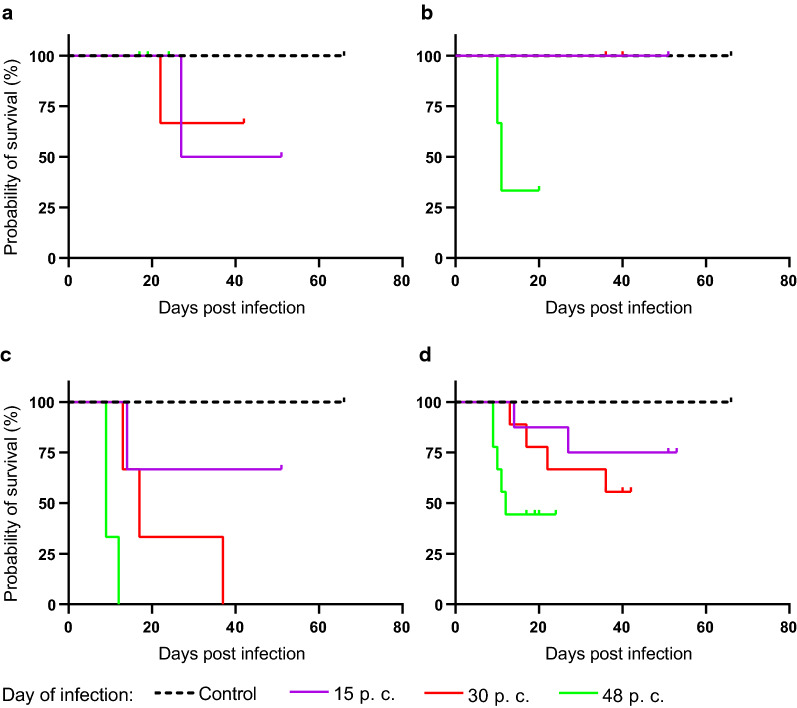
Impact of the infection duration on the dam survival rate. Kaplan-Meier survival rate of dams of the control and different infection groups. Animals were infected with 10 (**a**), 100 (**b**) and 500 (**c**) sporulated oocysts on gestation day 15 (purple solid line), 30 (red solid line) and 48 (green solid line). Animals of the control group (black dashed line) were administered PBS. The end of the trial for a given dam was defined by death, euthanasia due to a critical health score or abortion or by euthanasia on the day of birth. Merged data of all infection days, irrespective of the day of gestation (**a–c**) are shown in (**d**). Statistically significant differences are as follows: (**c**) *P* < 0.05, comparing control and 30; *P* < 0.05, comparing control and 48; *P* < 0.05, comparing 15 and 48; *P* < 0.05, comparing 30 and 48; (**d**) *P* < 0.5, comparing 15 and 48

All three dams of the control group survived until delivery (Figs. 2 and 3). For 11/26 (42%) of the remaining dams of the infection groups, the trial ended prematurely. Of these 11 dams, 3 died, 2 were killed because of a critical health score (> 20% body weight loss, severe central nervous symptoms including ataxia, somnolence, stupor, and severe algesia), and 6 were killed following abortion. For the remaining 15 (58%) dams of the infection groups, the trial ended on the day of delivery.

When the specific impact of the infection dose on the survival rate of all dams was investigated (Fig. 2), we noticed that the dose level was significantly related to the probability of survival. Administration of the highest dose, i.e. 500 oocysts, resulted in significantly (Additional file 2: Table S3) earlier loss and lower survival rate of the dams compared with administration of 10 oocysts (Fig. 2d). Moreover, administration of 100 oocysts leads to an earlier loss of dams than infection with 10 oocysts, although this difference was not statistically significant (Fig. 2d). When further taking the time of infection into account, administration of 500 oocysts on gestation day 48 resulted in a significantly (Additional file 2: Table S3) earlier loss and lower survival rate of dams than infection with 10 oocysts at the same gestational day (Fig. 2c). Again, administration of 100 oocysts on gestation day 48 leads to an earlier, although not statistically significant, loss and lower survival rate of dams than infection with 10 oocysts (Fig. 2c). A similar trend was observed for gestation days 15 and 30 with administration of 500 oocysts resulting in earlier death or euthanasia of the respective dams compared with those of lower infection doses; however, these differences were not statistically significant (Fig. 2a, b).

When analyzing the specific impact of the infection time point on the survival rate of all dams, we found a statistically significant correlation between gestational stage at infection and the probability of survival (Fig. 3). Infection in the third trimester of gestation, i.e. on gestation day 48, resulted in a significantly (Additional file 2: Table S4) earlier death or euthanasia compared with infection on day 15 of gestation (Fig. 3d). Moreover, infection on day 30 leads to an earlier loss and lower survival rate of dams than infection on day 15 of gestation, although this difference was not significant (Fig. 3d). Taking the infection dose into account, we found that infection with 500 oocysts on gestation day 48 resulted in a significantly (Additional file 2: Table S4) earlier loss and a lower survival rate of dams in comparison with infection on day 15 or 30 of gestation. Again, infection with 500 oocysts on gestation day 30 led to an earlier, although not statistically significant, loss and a lower survival rate than infection on gestation day 15 (Fig. 3c). For dams infected with 10 and 100 oocysts, no clear trend for the relationship between the infection time point and the probability of survival was observed (Fig. 3a, b).

Together, our data indicate that the probability of survival of dams infected with *T. gondii* highly depends on the dose and the gestational day at infection. Specifically, we show that higher infection doses, i.e. 500 oocysts, result in earlier loss and lower survival rate of dams than lower infection doses, i.e. 10 oocysts. Importantly, the majority of dams (78%) infected with the highest dose, and for which the trial ended prematurely, died or were euthanized because of a critical health score (Table 2). Moreover, our data show that an infection at later time points, i.e. third trimester of gestation, led to an earlier loss of dams compared with an infection occurring at earlier time points, i.e. first trimester of gestation. Notably, the majority of dams (80%) infected on gestation day 48 and for which the trial ended prematurely were euthanized prematurely following abortion (Table 2).

### Impact of the infection time point and the infection dose on the fate of the offspring

**Fig. 4:**
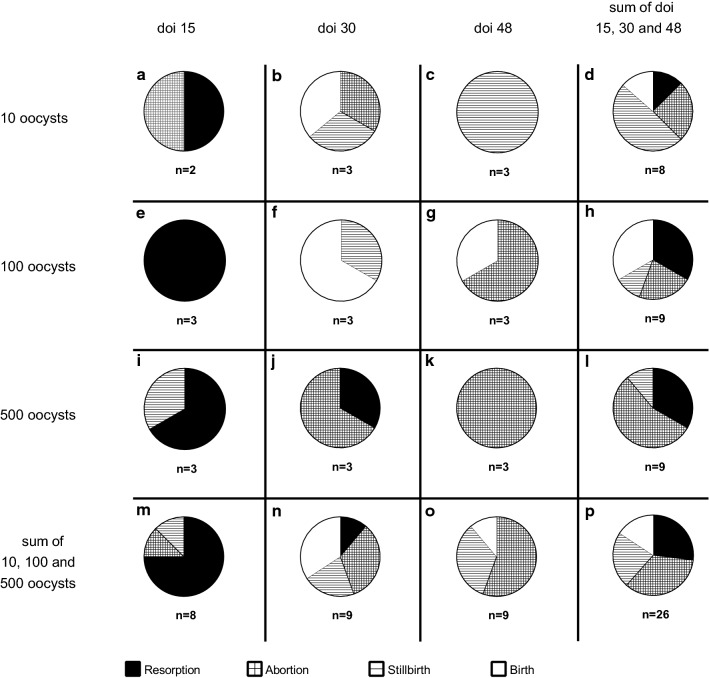
Impact of the infection dose and infection duration on the fate of offspring. Pie charts of the individual infection groups showing the proportional outcome (resorption, abortion, stillbirth, birth) of the respective litters. In case of dam no. 5 and 6 (Table 2), in which different events within the litters (e.g. birth and stillbirth) were observed, the outcome with the highest proportion was included in the analysis. doi, day of infection

We next investigated the fate of the offspring of each dam (Fig. 4). All three dams of the control group gave birth to asymptomatic offspring (Table 2). Of all 26 litters obtained from the infection groups, 34% of the offspring were aborted, 27% were resorbed, 23% were stillborn, and 16% were born asymptomatic (Fig. 4p). When analyzing the specific impact of the infection time point on the fate of the offspring, we found the highest survival rate for the offspring of dams infected on day 30 of gestation (Fig. 4n). Notably, infection of dams during the first trimester, i.e. on day 15 of gestation, ended lethally for 100% of the litters with the majority of offspring being resorbed during pregnancy (Fig. 4m). Moreover, infection of dams in the third trimester, i.e. on day 48 of gestation, ended lethally for the majority of litters with a very high proportion of stillbirth or abortion (Fig. 4o). Specifically, inoculation of 10 oocysts on gestation day 48 always resulted in stillbirth (Fig. 4c), whereas infection with 500 oocysts on gestation day 48 led to the abortion of all offspring (Fig. 4k).

When analyzing the specific impact of the infection dose on the fate of the offspring, we found all four possible outcomes, i.e. resorption, abortion, stillbirth and birth, to be present when dams were infected with 10 and 100 oocysts (Fig. 4d, h). The highest survival rate of the offspring was found when dams were infected with 100 oocysts (Fig. 4h). Notably, infection with the highest dose, i.e. 500 oocysts, ended lethally for 100% of the litters with the overwhelming majority of offspring being resorbed or aborted during pregnancy (Fig. 4l). Again, administration of 500 oocysts on day 15 of gestation (Fig. 4i) resulted in a higher proportion of fetal resorption compared with that of later gestation days (Fig. 4j, k).

Together, our data suggest that the survival rate of the offspring of dams infected with *T. gondii* depends on the dose and time point of infection. The highest survival rate of the offspring was observed when dams were infected with 100 oocysts on day 30 of gestation. However, administration of 500 oocysts, irrespective of the infection time point, and infection on day 15 of gestation, irrespective of the infection dose, ended lethally for 100% of the offspring.

### Impact of infection duration and infection dose on *T. gondii* DNA loads in the offspring

To investigate the impact of a *T. gondii* infection on *T. gondii* DNA loads in the offspring, various offspring tissues and organs were analyzed for the presence and amount of *T. gondii* DNA by qPCR using a *T. gondii*-specific 529-bp repeat element. Of all offspring (*n* = 53) obtained from all 26 litters, 7 (13%) delivered by three different dams infected with 10 or 500 oocysts on day 15 or 30 of gestation (Table 2, no. 2, 21 and 22) were severely autolytic and could not be used for qPCR analysis. In four (8%) offspring of two different dams infected with 500 oocysts on day 30 or 48 of gestation (Table 2, no. 24 and 25), *T. gondii* DNA was not detected in any of the offspring organs analyzed, i.e. brain, heart, liver, lung, spleen or femoral muscles, by qPCR (Table 2). Hence, the infection of these offspring was considered unsuccessful, and the offspring were excluded from the further analysis of this study. Given that the offspring of dams infected on day 15 of gestation, for which a *T. gondii* infection was verified, were either resorbed or severely autolytic (Table 2), qPCR data of this infection time point were not available, and the impact of a *T. gondii* infection in the first trimester of gestation on the *T. gondii* DNA loads in the offspring could not be analyzed. Of the remaining 42 (79%) offspring, *T. gondii* DNA was detected in all brain samples (Table 3). Moreover, in ≥ 60% of offspring samples obtained from the heart and femoral muscles, *T. gondii* DNA was present (Table 3). Among the offspring parenchymatous organs, *T. gondii* DNA was detected in 67% of all liver samples, whereas the percentage of lung and spleen samples containing *T. gondii* DNA was ≤ 50% (Table 3).

**Table 3:** Individual qPCR results for each offspring separated by organ or tissue

Oocyst dose	Day of inoculation	Litter no.	Offspring	Brain	Heart	Liver	Lung	Spleen	Muscle
10	30	4	A	+	−	−	−	−	−
10	30	5	A	+	−	+	−	−	−
10	30	5	B	+	−	−	+	IH	+
10	30	5	C	+	−	−	+	−	+
10	30	6	A	+	−	−	−	−	−
10	30	6	B	+	+	IH	IH	−	−
10	30	6	C	+	+	IH	IH	−	−
10	30	6	D	+	−	−	−	−	−
10	48	7	A	+	+	+	IH	IH	+
10	48	7	B	+	+	+	−	−	+
10	48	8	A	+	+	+	IH	+	+
10	48	8	B	+	+	+	+	+	+
10	48	8	C	+	+	+	IH	+	+
10	48	8	D	+	+	+	+	+	+
10	48	9	A	+	+	+	IH	+	+
10	48	9	B	+	+	+	IH	+	+
10	48	9	C	+	+	+	IH	IH	+
10	48	9	D	+	+	+	IH	+	+
100	30	13	A	+	−	−	IH	−	−
100	30	13	B	+	−	−	IH	−	+
100	30	13	C	+	+	−	IH	−	−
100	30	13	D	+	−	+	IH	−	+
100	30	14	A	+	−	−	+	IH	+
100	30	14	B	+	+	+	IH	−	+
100	30	15	A	+	+	−	IH	−	−
100	30	15	B	+	+	−	−	−	+
100	30	15	C	+	+	−	IH	IH	+
100	48	16	A	+	−	+	IH	−	IH
100	48	16	B	+	+	+	IH	IH	IH
100	48	17	A	+	+	+	IH	-	IH
100	48	17	B	+	+	+	IH	+	IH
100	48	17	C	+	−	+	IH	+	IH
100	48	18	A	+	+	+	IH	−	+
100	48	18	B	+	+	+	IH	−	+
100	48	18	C	+	+	−	IH	−	+
100	48	18	D	+	+	+	IH	−	+
500	30	24	B	+	+	+	IH	+	−
500	48	26	A	+	+	+	IH	+	−
500	48	26	B	+	+	IH	IH	IH	−
500	48	26	C	+	+	+	IH	+	−
500	48	26	D	+	+	+	IH	IH	−
500	48	27	A	+	+	+	+	+	+
Positivity rate				100%	71%	67%	50%	38%	62%

“ + ” represents a positive result, “-” a negative result. No result could be generated for “IH" due to PCR inhibition

*no*. number, *IH* inhibition

**Fig. 5:**
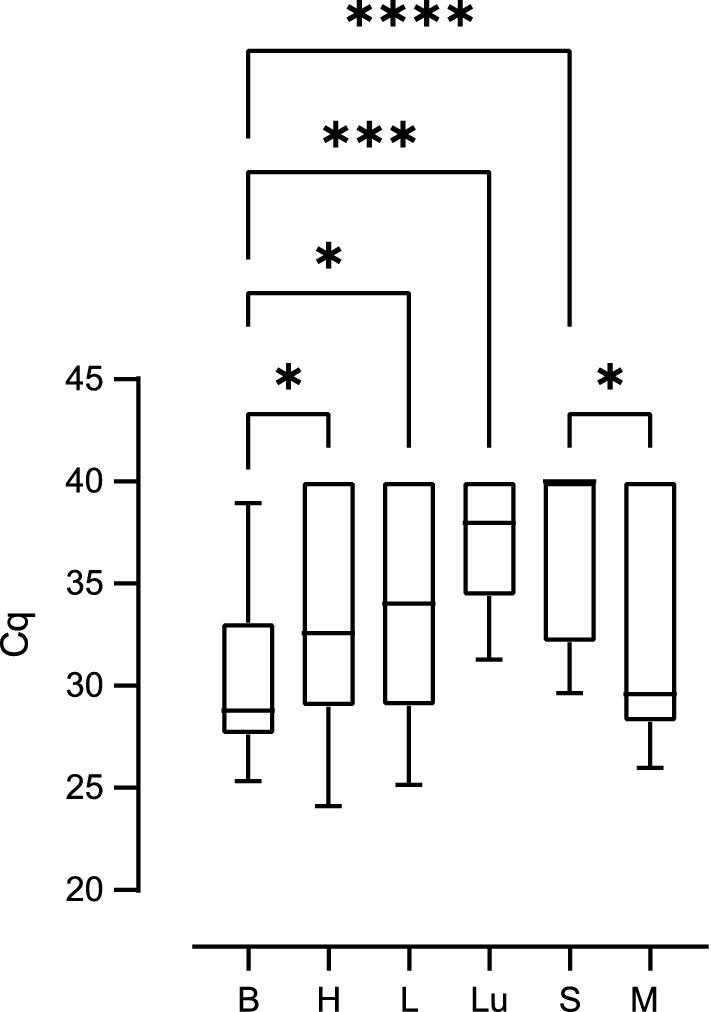
Impact of *T. gondii* infection on Cq values for *Toxoplasma gondii* DNA in the offspring. Cq values for *T. gondii* DNA extracted from tissues and organs of offspring obtained from dams infected with 10, 100 or 500 oocysts on gestation day 15, 30 or 48. Solid lines indicate comparison between offspring tissue or organs. *P < 0.05; **P < 0.01; ****P < 0.0001. *B* brain, *H* heart, *L* liver, *Lu* lung, *S* spleen, *M* femoral muscles

**Fig. 6:**
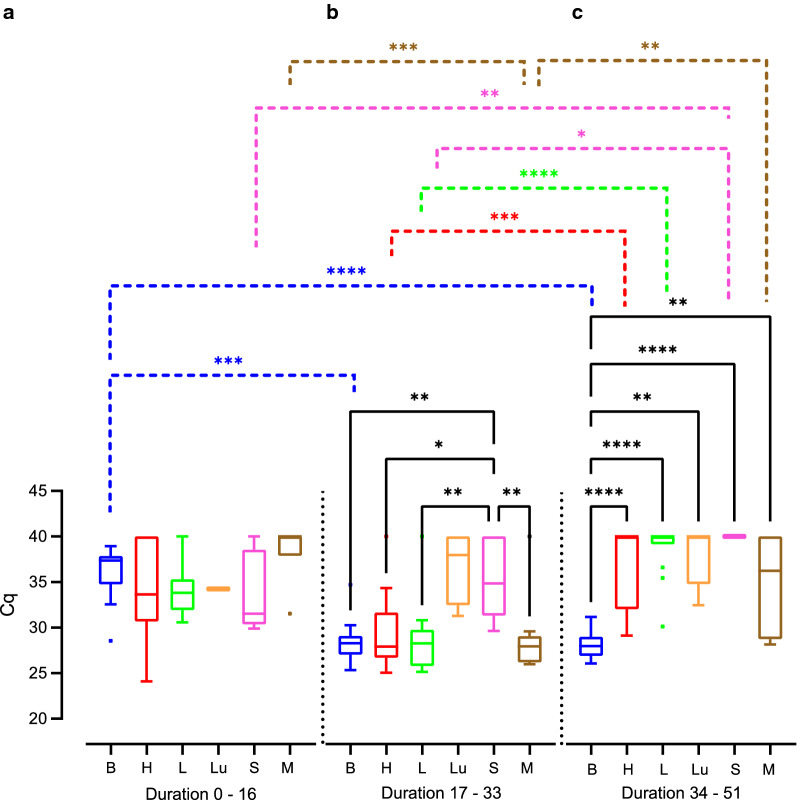
Impact of the infection duration on Cq values for *Toxoplasma gondii* DNA in the offspring. Cq values for *T. gondii* DNA extracted from tissues and organs of offspring obtained from dams infected for different durations. Solid lines indicate comparison of offspring organs obtained from dams infected for the same inoculation duration. Dashed lines indicate comparison of the same organ between different offspring survival times. *P < 0.05; **P < 0.01; ***P < 0.001; ****P < 0.0001. *B* brain, *H* heart, *L* liver, *Lu* lung, *S* spleen, *M* femoral muscles

We next compared the Cq values obtained by qPCR as a proxy for *T. gondii* DNA load between the different tissue and organ samples of all different infection groups. Cq values were significantly lower in the offspring brain compared with that of heart, lung and spleen (Fig. 5, Additional file 2: Table S5, Additional file 2: Table S6). No significant difference in the Cq values was observed when offspring heart, liver and lung were compared (Fig. 5). Since the infection duration varies greatly between the different infection groups, even when dams were infected on the same gestational day, we focused our analysis on the impact of the actual duration dams were infected, irrespective of the infection time point, on the amount of *T. gondii* DNA in the offspring tissue and organ samples (Fig. 6). For this, the infection duration of dams was subdivided into three different groups: 0–16, 17–33 and 34–51 days.

When comparing Cq values between different tissues and organs within the same group of duration of infection (Fig. 6), our data show that *T. gondii* infection of dams for a duration of 34–51 days results in significantly lower Cq values in the offspring brain compared with those of heart, liver, lung, spleen and femoral muscles (Fig. 6c, Additional file 2: Table S5, Additional file 2: Table S7). Similarly, when dams were infected for a shorter duration, i.e. 17–33 days, Cq values in the offspring brain were significantly lower compared with those of spleen; however, they did not differ markedly from values in the heart, femoral muscles, lung and liver for the same infection duration of the dam (Fig. 6b, Additional file 2: Table S5, Additional file 2: Table S7). No significant differences between the Cq values of all offspring tissues and organs analyzed were observed when dams were infected for 0–16 days (Fig. 6a).

When comparing Cq values of the same tissue or organ between different groups of infection durations, Cq values in the offspring heart, liver, spleen and femoral muscles were significantly higher when dams were infected for 34–51 days (Fig. 6c, Additional file 2: Table S5, Additional file 2: Table S7) compared to those infected for a shorter duration, i.e. 17–33 days, or in case of the spleen 0–16 days (Fig. 6a, b). In contrast, median Cq values were found to decrease in the offspring brain with increasing infection duration of a dam. Specifically, Cq values in the offspring brain were significantly lower when dams were infected for 34–51 or 17–33 days (Fig. 6b, c, Additional file 2: Table S5, Additional file 2: Table S7) than in the offspring brain of dams infected for 0–16 days (Fig. 6a), respectively.

**Fig. 7:**
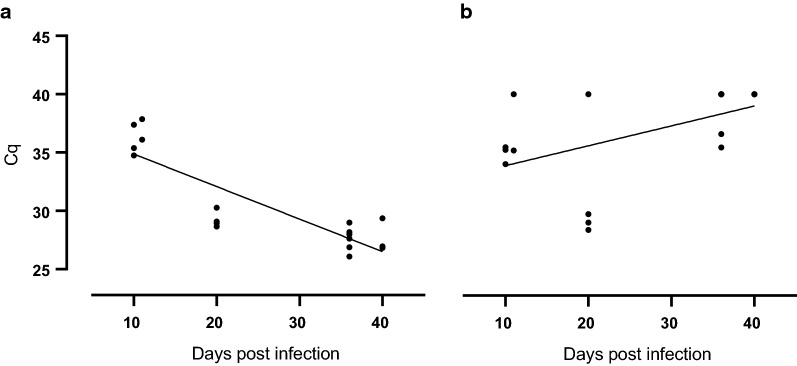
Relationship between duration of infection and Cq values for *Toxoplasma gondii* DNA in the offspring. Cq values of offspring brain (**a**) and liver (**b**) obtained from dams infected with 100 oocysts as determined by *T. gondii* qPCR. For offspring brain, the regression was characterized by *P* < 0.0001, R^2^ = 0.7464 and f (x) = − 0.2786x + 37.65. For offspring liver, the regression was characterized by *P* < 0.05, R^2^ = 0.2556 and f (x) = 0.1702x + 32,20. Data were analyzed using the least squares method

Together, our data show that the duration of *T. gondii* infection impacts on the amount of *T. gondii* DNA in the offspring with longer infection durations of pregnant dams, i.e. 34–51 days, relating to lower Cq values, and thus obviously higher *T. gondii* DNA levels, in the offspring brain and to higher Cq values, and thus obviously lower *T. gondii* DNA levels in most of the offspring abdominal organs examined. This observation is further supported by the estimation of the relationship between the Cq value obtained in the offspring brain and liver and infection durations of dams as analyzed by regression analysis (Fig. 7, Additional file 2: Table S5, Additional file 2: Table S8). In particular, it shows that Cq values and infection duration are significantly negatively correlated (Fig. 7a). In contrast, in the offspring liver, Cq value and infection durations are significantly positively correlated (Fig. 7b).

**Fig. 8:**
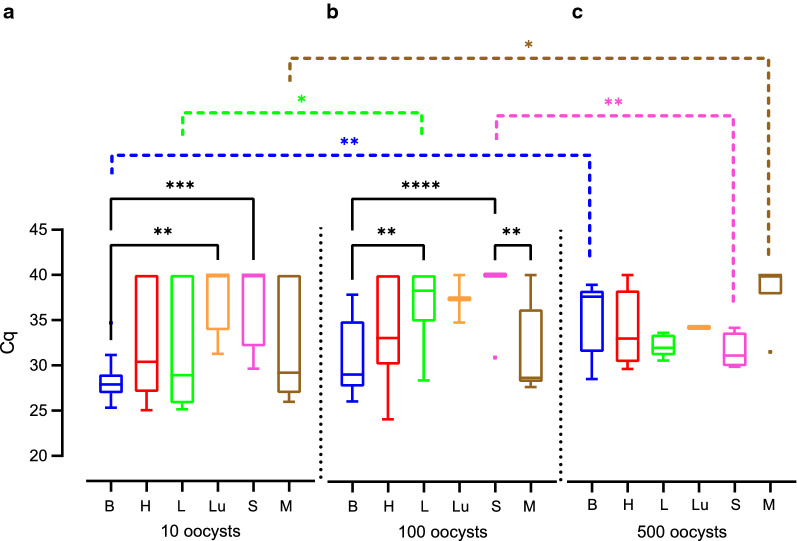
Impact of the infection dose on Cq values for *Toxoplasma gondii* DNA in the offspring. Cq values of different offspring tissue and organs obtained from dams inoculated with different *T. gondii* doses as detected by qPCR. Solid lines indicate comparison of different offspring organs obtained from dams infected with the same inoculation dose. Dashed lines indicate comparison of the same tissue and organ between different inoculation doses. *P < 0.05, **P < 0.01, ***P < 0.001; ****P < 0.0001. *B* brain, *H* heart, *L* liver, *Lu* lung, *S* spleen, *M* femoral muscles

We next analyzed the impact of the infection dose on the amount of *T. gondii* DNA in the offspring tissue and organ samples (Fig. 8). When comparing Cq values between different tissues and organs of offspring obtained from dams infected with the same infection dose, our data show that administration of 10 and 100 oocysts results in significantly lower Cq values in the offspring brain compared with those of spleen, lung or liver (Fig. 8a, b, Additional file 2: Table S5, Additional file 2: Table S9). No significant differences, except for spleen and femoral muscles infected with 100 oocysts, were observed when different tissues and organs were compared following an administration of 100 or 500 oocysts, respectively (Fig. 8b, c).

When comparing Cq values of the same tissue or organ between different infection doses, Cq values in the offspring brain were significantly higher when dams were infected with 500 oocysts (Fig. 8c, Additional file 2: Table S5, Additional file 2: Table S9) compared with those infected with lower infection doses, i.e. 10 and 100 oocysts (Fig. 8a, b), and Cq values in the offspring brain were significantly higher when dams were infected with 100 oocysts (Fig. 8b) compared with those infected with 10 oocysts (Fig. 8a). Moreover, significantly higher Cq values were observed in the offspring femoral muscles following an infection with 500 oocysts (Fig. 8c) and liver following an infection with 100 oocysts (Fig. 8c) compared with those infected with a lower infection dose, i.e. 10 oocysts (Fig. 8a). No significant differences between different infection doses were observed when all other organs were compared (Fig. 8).

**Fig. 9:**
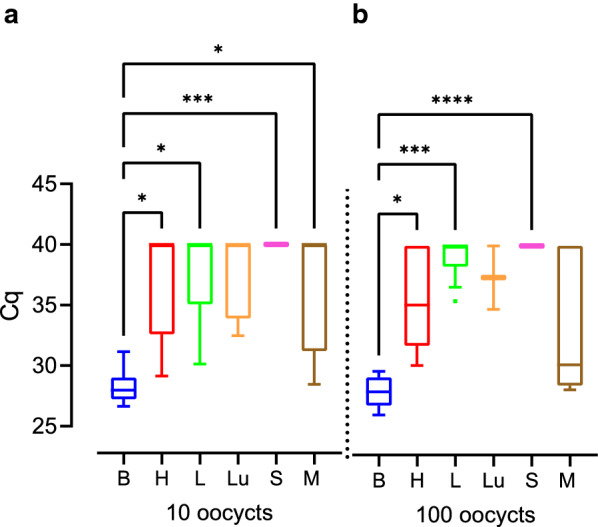
Impact of the infection dose on Cq values for *Toxoplasma gondii* DNA in the offspring. Cq values measured in various offspring tissues and organs, of which dams were infected for 34 to 51 days, with different *T. gondii* doses. Solid lines indicate comparison of different offspring organs obtained from dams infected with the same inoculation dose. Dashed lines indicate comparison of the same tissue and organ between different inoculation doses. *P < 0.05, **P < 0.01, ***P < 0.001, ****P < 0.0001. *B* brain, *H* heart, *L* liver, *Lu* lung, *S* spleen, *M* femoral muscles

When comparing Cq values of various tissues and organs of offspring obtained from dams infected for the same infection duration, i.e. 34–51 days, no significant differences were observed when 10 and 100 oocysts were administered (Fig. 9, Additional file 2: Table S5, Additional file 2: Table S10). This indicates that the *T. gondii* infection dose has less impact on the amount of *T. gondii* DNA level in the offspring.

Last, we investigated whether the amount of *T. gondii* DNA in the guinea pig offspring corresponded to its survival and compared Cq values of various tissue and organs of stillborn offspring and those born asymptomatic, all obtained from dams infected for the same infection duration, i.e. 34–51 days. This reveals that Cq values of offspring brain, liver, heart, lung, spleen and femoral muscle did not differ significantly between stillborn offspring and those born asymptomatic (Additional file 2: Table S11, Additional file 3: Figure S1), thus indicating that the amount of *T. gondii* DNA in the guinea pig offspring might not corresponds to survival.

### Impact of *T. gondii* infection on the integrity of the offspring brain

**Fig. 10:**
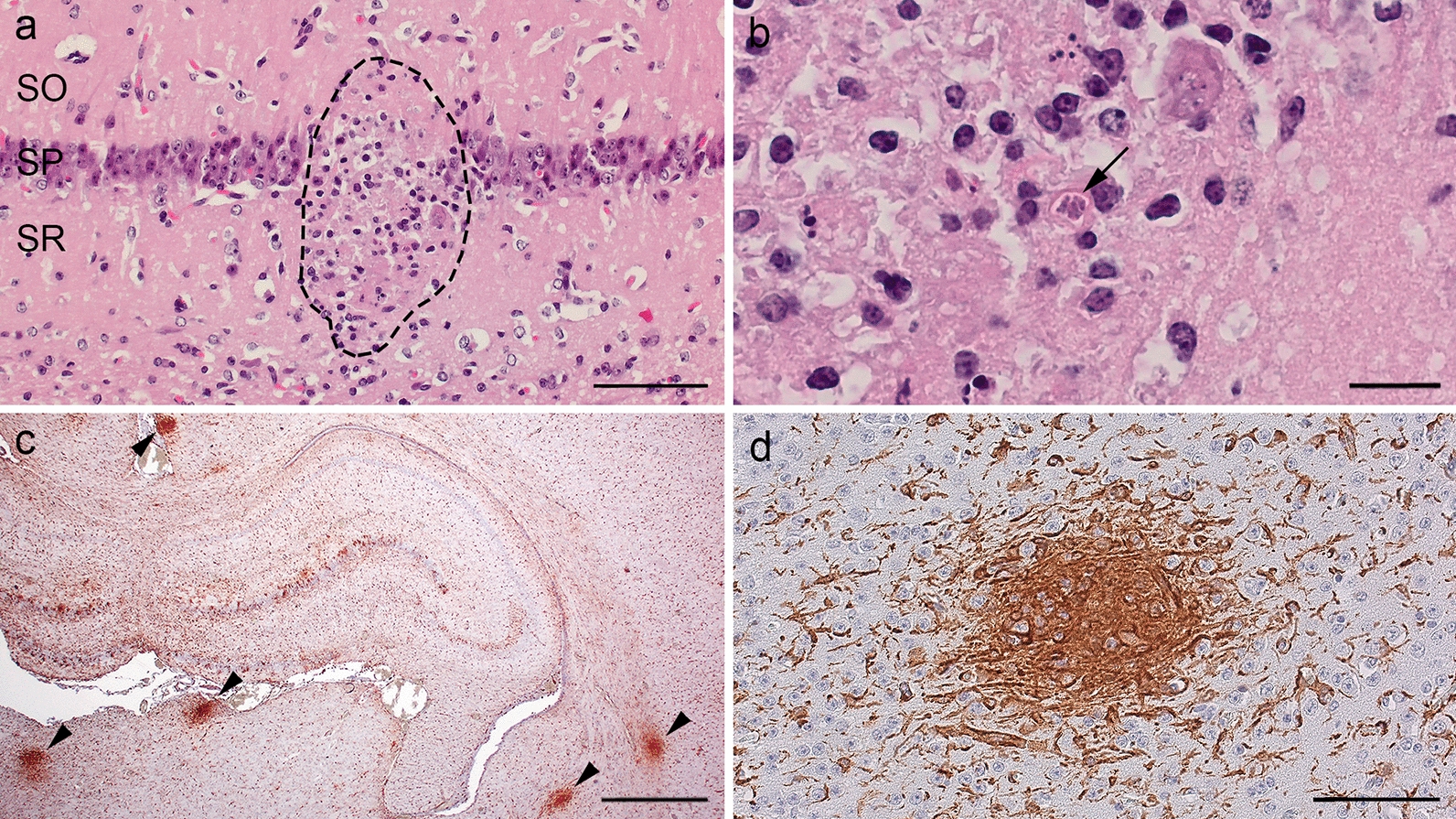
Histological examination of the offspring brain infected with *Toxoplasma gondii*. **a** A focal necrosis with microgliosis in the hippocampus stained with hematoxylin-eosin. Dashed lines indicate delineation of the focus. SO, stratum oriens; SP, stratum pyramidale; SR, stratum radiatum, scale bar = 50 µm. **b** A subset of **a** in higher magnification, scale bar = 10 µm. The arrow indicates intracytoplasmic tachyzoites. **c** An overview image of an IBA 1 immunohistochemical stain in the hippocampal region. Arrowheads indicate the IBA 1-positive foci, scale bar = 500 µm. **d** A subset of **c** in higher magnification (scale bar = 50 µm)

**Fig. 11:**
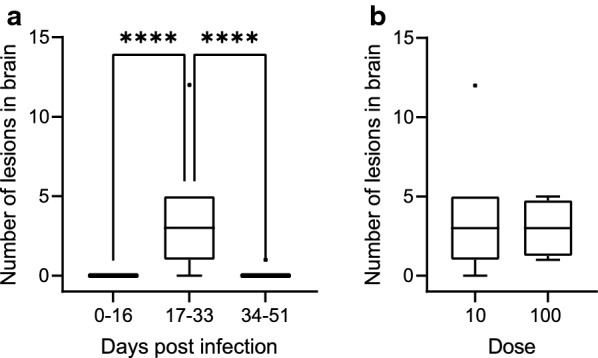
Impact of the infection duration and infection dose on the integrity of offspring brain. **a** The number of lesions detected per offspring brain in relation to the duration dams were infected (****P < 0.0001). **b** The number of lesions per offspring brain, of which dams were infected for 17 to 33 days, in relation to the infection dose. **a, b** Number of lesions was counted in the five brain sections of each brain. For details, see Methods

In a next step, we assessed the impact of *T. gondii* infection on the integrity of the offspring brain by pathohistological examination. For this, the brain of all offspring was cut coronally into five divisions of similar thickness and one section per brain division was stained with hematoxylin-eosin and an antibody for Iba1, a marker of microglia. In all offspring of the control group, no pathological changes including necrotizing necrosis lesions were detected in the brain. In contrast, in the offspring of the infection groups, focal or multifocal microgliosis and/or necrotizing encephalitis were present in numerous brains (Fig. 10). Moreover, within the center of some lesions, intracellular protozoal structures were detectable in intracellular vacuoles (Fig. 10b). A total of 50 microglia-associated lesions were quantified in the brain sections of all offspring analyzed. When comparing the number of lesions per offspring brain between dams infected for different durations, our data show that the number of lesions observed in the offspring brain of dams infected for 17–33 days was significantly higher than that of those obtained from dams infected for 0–16 and 34–51 days (Fig. 11a, Additional file 2: Table S12, Additional file 2: Table S13). When comparing the number of lesions per offspring brain between dams inoculated with different doses within the same duration of infection, i.e. 17–33 days, no significant differences could be observed (Fig. 11b, Additional file 2: Table S12, Additional file 2: Table S13).

Together, our data show that *T. gondii* infection affects the integrity of the offspring brain. Specifically, *T. gondii* infection of dams for 17–33 days results in a high abundance of nodular microgliosis in the offspring brain, whereas the infection dose seems to have little impact on the abundance of microglia-associated lesions in the offspring brain.

## Discussion

This project provides extensive contributions to establishing the guinea pig as a suitable animal model for (human) congenital toxoplasmosis. Since all dams became pregnant at least upon second mating and no abnormalities were found in either the pregnant or non-pregnant animals, we conclude that fertility and reproductive problems are unlikely to have interfered with the *T. gondii* infection results. By oral administration of *T. gondii* oocysts, we chose a natural route of infection for the guinea pig, because as an herbivorous animal it becomes infected almost exclusively via oocyst-contaminated food or water. Oocyst infection also plays an important role in humans, since up to 43–78% of human *T. gondii* infections are considered to be transmitted via oocyst-contaminated food or water [44, 45]. As this is the first study using the ME49 strain for congenital infections in guinea pigs, *T. gondii* oocyst doses were chosen based on previous studies using oocysts of ME49 strain for congenital infection in mice and adapted to account for the (approximately 20×) higher guinea pig body weight [46].

We show that the probability of seroconversion in *T. gondii*-infected pregnant guinea pigs strongly depends on the duration a dam is infected with its probability exceeding 50% after day 20 of infection. This is in line with previous studies in guinea pigs and other mammals showing seroconversion to usually occur between 9 and 21 days post-infection [32, 46–48]. Given that in almost all seronegative dams *T. gondii* was directly detected in at least one maternal organ using qPCR, it can be assumed that the time between infection and sampling in those dams was simply not long enough for specific antibodies to become detectable in the blood. In addition, our data indicate the dose of infection seems to have little impact on the probability of seroconversion in *T. gondii*-infected pregnant guinea pigs, which also agrees with previous findings in mice [46].

Similar to findings in guinea pigs and other mammalian species [21, 22, 46, 48], we found that the survival rate of *T. gondii*-infected dams strongly differs depending on the dose of infection. In general, it was found that increasing oocyst doses caused earlier offspring losses and lower survival rates of pregnant dams. Specifically, administration of the highest infection dose, i.e. 500 oocysts, results in a significantly earlier loss and lower survival rate of dams than infection with lower infection doses, i.e. 10 oocysts. In this regard, a potential relationship between the pregnancy and a low survival rate of dams cannot be excluded. As this is the first study using oocysts of ME49 strain for infection in guinea pigs, further studies including *T. gondii* infections of non-pregnant animals are needed to determine whether a low survival rate, especially after administration of 500 oocysts, is related to pregnancy or rather a general phenomenon in guinea pigs.

Moreover, we show that infection with 500 oocysts always ended lethally for the respective litters, with > 50% of offspring being aborted. A similar dose-dependent effect on the fate of the offspring has been previously observed in guinea pigs and other mammals including mice showing that higher *T. gondii* doses generally result in a smaller litter size [21, 46, 48]. Additionally, the fate of the offspring seems to depend on the gestational stage at which the dam is infected. In particular, a *T. gondii* infection of dams during the first trimester, i.e. on day 15 of gestation, ended lethally for all litters with most offspring being resorbed during pregnancy, as has been shown in humans [19, 49]. Intriguingly, infection of dams in the third trimester, i.e. on day 48 of gestation, ended lethally for most litters with a very high proportion of offspring stillbirth or abortion. Similarly, a previous study in guinea pigs showed that *T. gondii* infection at the end of the second trimester of gestation leads to significantly higher offspring losses compared to infection in the first trimester of gestation [21]. However, our findings contrast with findings in humans, in which infection during the third trimester of gestation often remains asymptomatic for the fetus [49, 50]. One possible scenario for this difference might be that *T. gondii* infiltrates and disrupts the placenta more severely in guinea pigs compared to humans upon an infection in the third trimester of gestation. Given that the placental tissue was consumed by the dams immediately after abortion, an examination of the placental tissue was not possible in this study. We therefore recommend this analysis to be part of further studies.

Furthermore, we show that the duration a pregnant dam is infected strongly impacts on the amount of *T. gondii* DNA in the guinea pig offspring. Specifically, a *T. gondii* infection of dams for < 17 days appears to result in similar *T. gondii* DNA loads in various offspring organs and tissues, namely brain, liver, spleen, heart, lung and femoral muscles. However, an infection of pregnant dams for > 34 days seems to correspond to higher *T. gondii* DNA levels in the offspring brain but lower *T. gondii* DNA levels in all body cavity organs examined. This indicates that *T. gondii* initially replicates in various offspring tissues and organs during the acute stage of infection after which it tends to disappear from most of the body cavity organs to accumulate in the brain. Moreover, our findings support the notion that the brain is considered the preferred host organ of the permanent stages of the parasite in mammals including guinea pig and human [22, 46, 51, 52].

Interestingly, it was found that administration of high *T. gondii* infection doses, i.e. 500 oocysts, might result in lower *T. gondii* DNA levels in the offspring brain compared to low doses, i.e. 10 oocysts. Considering that higher infection doses significantly reduce offspring survival time, lower *T. gondii* DNA levels in the offspring organ, i.e. the brain, upon administration of high infection doses might reflect the fact that the duration of infection was simply not long enough for the parasite to accumulate in various offspring organs. Indeed, for all dams, which were administered 500 oocysts the trial ended within the first 16 days after infection. Hence, when Cq values of various tissue and organs of offspring obtained from dams infected were compared for the same infection duration, no significant differences were observed between the different oocyst infection doses administered. Thus, our data suggest that the *T. gondii* infection dose seems to have little, if any, influence on the amount of *T. gondii* DNA in the guinea pig offspring.

The duration a pregnant dam is infected also affects the integrity of the offspring brain. In particular, infection of dams for 17–33 days results in significantly increased nodular microgliosis in the offspring brain compared to shorter periods of infection, thus demonstrating that focal cell lesions caused by tachyzoites trigger a microglia immune response in the acute phase of *T. gondii* infection [16]. Intriguingly, the abundance of microglia-associated lesions was significantly lower in the offspring brain when dams were infected for > 34 days compared to 17–33 days. This potentially reflects the fact that, once the infection is controlled by the immune system, tachyzoites transform into bradyzoites, which are able to escape the host immune response [16, 53]. Again, the infection dose seems to have little impact on the abundance of microglia-associated lesions and thus the integrity of the offspring brain.

Taken together, our data show that the ME49 strain of *T. gondii* is vertically transmitted in the guinea pig and infects the unborn offspring, thus demonstrating its suitability for studying congenital toxoplasmosis. Based on our data, infection of pregnant dams with 10–100 T*. gondii* oocysts in the second trimester of gestation is suitable for investigating the course of *T. gondii* infection in the fetus and postnatal animal, specifically the *T. gondii*-associated pathological changes, underlying pathomechanisms and immune responses in various offspring tissues and organs including the brain. Moreover, infection of pregnant guinea pigs in the first trimester of gestation is particularly suited to investigate the pathogenesis of fetal resorption and to examine treatment options to prevent the dramatic outcome of toxoplasmosis during the early stage of infection in pregnant women. Further studies including varying *T. gondii* doses and a larger number of animals are needed to obtain detailed data on the impact of a congenital *T. gondii* infection on the survival of dams and their offspring as well as on parasite loads in tissues and organs of prenatally infected offspring.

## Conclusions

This study demonstrates that the ME49 strain of *T. gondii* is suitable for studying congenital toxoplasmosis. Specifically, it reveals that the *T. gondii* infection dose and the gestational stage at infection markedly influence the survival rate of dams and the fate of the offspring. Moreover, the duration of infection substantially impacts the seroconversion rate of dams, the *T. gondii* DNA loads in the offspring and the integrity of offspring brain. Together, this work contributes significantly to establishing the guinea pig as a suitable animal model for human congenital toxoplasmosis, which can be used to solve scientific questions of high medical significance, i.e. regarding the pathogenesis, immunological responses, efficacy of vaccines and drugs as well as the establishment of practice-relevant diagnostic markers of congenital *T. gondii* infection in humans.

## Supplementary Information

**Additional file 1: Table S1.** Scoring system used to evaluate the clinical condition of guinea pigs.

**Additional file 2: Table S2.** Logistic regressions of the probability of seroconversion depending on the duration of infection and the infectious dose as shown in Fig. 1. **Table S3.** Individual results of the multiple Gehan-Breslow-Wilcoxon tests as shown in Fig. 2. **Table S4.** Individual results of the multiple Gehan-Breslow-Wilcoxon tests as shown in Fig. 3. **Table S5**. Cq values of individual organs of each offspring. **Table S6.** Individual results of the Kruskal-Wallis tests as shown in Fig. 5. **Table S7.** Individual results of the Kruskal-Wallis tests as shown in Fig. 6. **Table S8.** Individual results of the simple linear regressions of brains and hearts as shown in Fig. 7. **Table S9.** Individual results of the Kruskal-Wallis tests as shown in Fig. 8. **Table S10.** Individual results of the Kruskal-Wallis tests as shown in Fig. 9. **Table S11.** Individual results of the Mann-Whitney tests as shown in Additional file 3: Figure S1. **Table S12.** Number of histopathologically determined lesions in the brain of each offspring. **Table S13.** Individual results of the Kruskal-Wallis and Mann-Whitney tests as shown in Fig. 11.

**Additional file 3: Figure S1.** Impact of the Cq value on the fate of offspring. Cq values measured in various offspring tissues and organs, of which dams were infected for 34 to 51 days, with different *T. gondii* doses. No significant differences were found between the stillborn offspring and the offspring born asymptomatic.* SB* stillbirth,* BA* born alive.

## Data Availability

The data generated or analyzed during this study are included in this published article or can be obtained from the corresponding author on request.

## Abbreviations

B: Brain
bp: Base pair
bw: Body weight
Cq: Quantification cycle
DNA: Deoxyribonucleic acid
doi: Day of infection
F: Fetal
H: Heart
IC: Internal control
IH: Inhibition
L: Liver
Lu: Lung
M: Femoral muscles
N: Neonatal
PBS: Phosphate-buffered saline
p. c.: Post-conceptionem
PCR: Real-time quantitative polymerase chain reaction
qPCR: Real-time quantitative polymerase chain reaction
S: Spleen
SB: Stillbirth
BA: Born alive
SO: Stratum oriens of hippocampus
SO: Stratum pyramidale of hippocampus
SR: Stratum radiatum of hippocampus
*T. gondii*: *Toxoplasma gondii*
TgREP-529: 529-Base pair repetitive element of *T. gondii*
