# Supplementary material for: Establishment and validation of a guinea pig model for human congenital toxoplasmosis
Source: Parasit Vectors. 2021 Aug 6;14:389. doi: 10.1186/s13071-021-04890-4 (PMC8344189; doi:10.1186/s13071-021-04890-4)
Supplement: Supplementary file 1 — Additional file 1: Table S1. Scoring system used to evaluate the clinical condition of guinea pigs. [file 13071_2021_4890_MOESM1_ESM.docx]

**Additional file 1: Table S1.** Scoring system used to evaluate the clinical condition of guinea pigs.

| **Criteria** | **0** | **1** | **2** | **3** |
| --- | --- | --- | --- | --- |
| **Activity** | normal | isolated, abnormal posture | inactive or hyperactive | moribund |
| **Movement, gait** | normal | slightly uncoordinated or abnormal gait | uncoordinated or no longer fully applies limb or aversion to movement | swaying or does not sit up limb or paralysis |
| **Hair coat** | normal (smooth, shiny, close-fitting) | brittle | unkempt, wounds, loss of hair | bleeding or infected wounds, massive hair loss, automutilation |
| **Nutritional status** | normal (ribs palpable, abdomen barrel-shaped, no gluteal and axillary fat pads) | thin | loss of body fat | loss of muscle mass |
| **Water intake** | normal (50-100 ml/kg bw) | decreased or increased over 24 h | decreased or increased over 48 h | permanent drinking or no water intake over 24 h |
| **Food intake** | normal (30-50 g pellets/kg bw) | decreased or increased over 24 h | decreased or increased over 48 h | obese or inappetence over 48 h |
| **Urine** | normal (straw-yellow) |  | abnormal color / quantity | no urine output over 24 h or wet perineum |
| **Feces** | normal (dry, clumped) | mushy, moist | diarrhea or decreased fecal output | diarrhea over 24 h, bloody admixtures, no defecation over 24 h |
| **Breathing** | normal, 100-150 breaths/min | elevated, flat | elevated, abdominal | dyspnea, irregular, cyanotic colored mucous membranes |
| **Vocalization** | normal, minor with manipulation | none in case of manipulation | high in case of manipulation |  |
| **Dehydration** | none | reduced skin elasticity | skin fold persists | skin fold remains, sunken eyes |
| **Eyes** | normal | dry, dull | eye discharge | lids stuck together, hemophthalmos, exophthalmos |
| **Nose** | normal | dried out | minor nasal discharge | high-grade discharge, stuck |
| **Mucosal color** | pale pink |  | reddened or pale | icteric, high-grade redness or cyanotic |
| **Vagina** | dry, no discharge | swollen | clear discharge | cloudy, purulent or bloody discharge |

If the additive score is greater than 3, the veterinarian and project manager will be informed, as well as the animals will be clinically examined in detail daily and additionally clinically observed at least once. In addition, appropriate measures are taken if necessary. Depending on the clinical manifestations, these include further diagnostic measures (e.g. weighing, microbiological examinations, antibiogram, etc.), keeping a lower number of animals per cage unit (stress reduction), symptomatic or causal therapy (depending on the drug also study exclusion) or euthanasia.

If the additive score is greater than 5, painful conditions are assumed and 1.33 mg/ml metamizole sodium is added to the drinking water.

If a score of 2 is achieved in the dehydration category, 60 ml/kg bw and a score of 3, 100 ml/kg bw of a 0.9% sodium chloride solution is applied subcutaneously.

If an additive score of at least 2 is reached in the following categories: activity, hair coat, nutritional status, water and feed intake, then social stress conditions are assumed, and the animal is separated into an additional cage.

**Humane endpoint:**

- from an additive score of 10,
- assessment of two criteria with a score of 3,
- weight loss of 15% or more,
- ascites,
- abortion,
- inability to move,
- blindness,
- central nervous deficits
- or the presence of even individual clinical manifestations of corresponding severity (with or without connection to the scoring system presented or to the exposure of the animals to the actual animal experiment) the affected animals are euthanized. This is done according to the assessment of the responsible veterinarian.
