# Supplementary figures and images for: Establishment and validation of a guinea pig model for human congenital toxoplasmosis
Source: Parasit Vectors. 2021 Aug 6;14:389. doi: 10.1186/s13071-021-04890-4 (PMC8344189; doi:10.1186/s13071-021-04890-4)

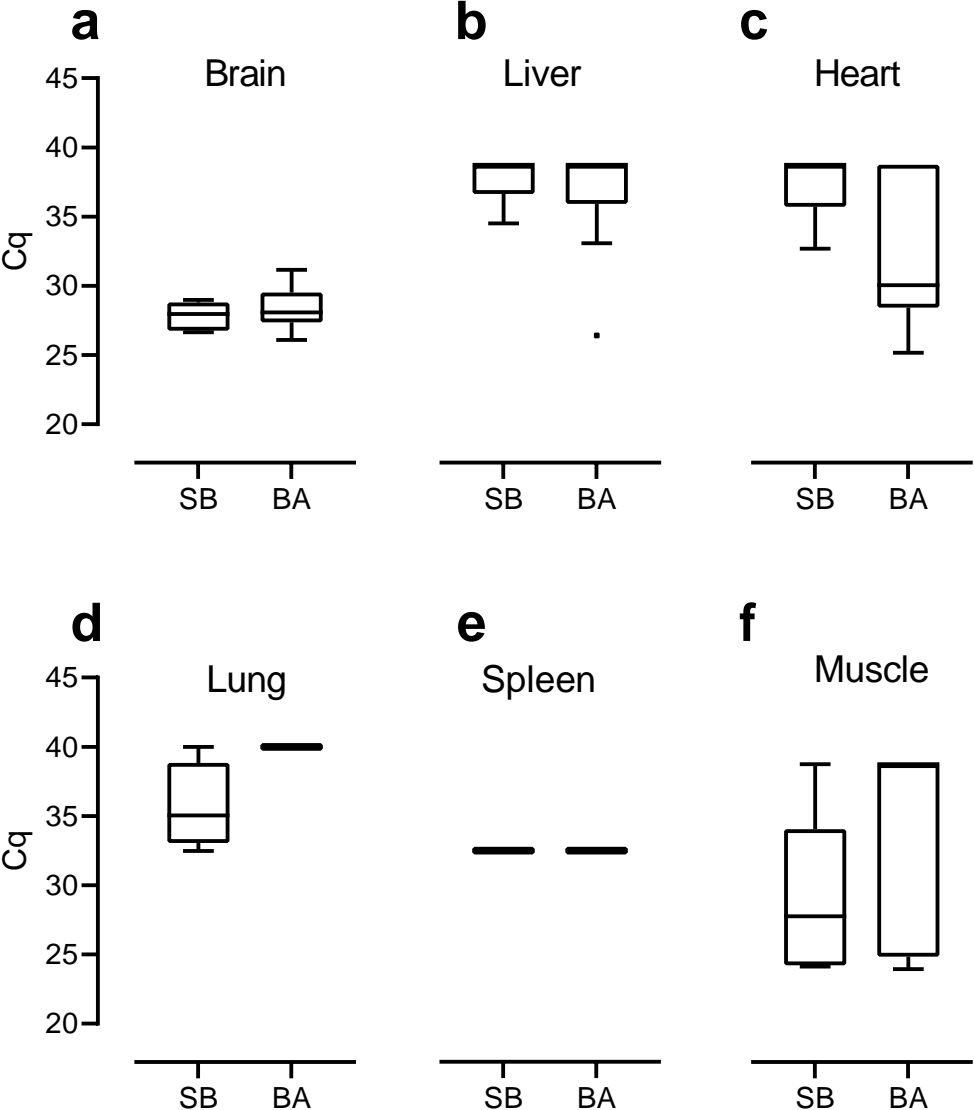

Supplement: Supplementary file 3 — Additional file 3: Figure S1. Impact of the Cq value on the fate of offspring. Cq values measured in various offspring tissues and organs, of which dams were infected for 34 to 51 days, with different T. gondii doses. No significant differences were found between the stillborn offspring and the offspring born asymptomatic. SB stillbirth, BA born alive. [file 13071_2021_4890_MOESM3_ESM.pdf]
